# Meckel's diverticulum: an exceptional cause of vesicoenteric fistula: case report and literature review

**DOI:** 10.11604/pamj.2013.15.9.2440

**Published:** 2013-05-06

**Authors:** Mahdi Bouassida, Mohamed Mongi Mighri, Khaled Trigui, Mohamed Fadhel Chtourou, Selim Sassi, Bilel Feidi, Fathi Chebbi, Khaled Bouzaidi, Hassen Touinsi, Sadok Sassi

**Affiliations:** 1 Department of surgery, Mohamed Tahar Maamouri Hospital, Mrazga 8000 Nabeul; 2 Department of radiology, Mohamed Tahar Maamouri Hospital, Mrazga 8000 Nabeul, Tunisia

**Keywords:** Meckel's diverticulum, vesicoenteric fistula, surgery

## Abstract

Meckel's diverticulum is the most common congenital malformation of the gastrointestinal tract. It can cause complications in the form of ulceration, hemorrhage, intussusception, intestinal obstruction, perforation and, very rarely, vesicodiverticular fistulae as noted in six previously reported cases. 66-year-old woman was presented with an enterovesical fistula. Exploratory laparotomy revealed a vesico-diverticular fistula resulting from a perforated Meckel′s diverticulum. Pathologic examination revealed that the diverticulum did not contain ectopic gastric or pancreatic tissue. The patient underwent a diverticulectomy and had an uneventful postoperative course. Unlike four of the six previously reported cases, our patient had no coexisting bowel or bladder disease occurring with her vesico-diverticular fistula. Conclusion: This is only the third reported case of a vesico-diverticular fistula resulting from a perforated Meckel′s diverticulum that did not contain ectopic tissue.

## Introduction

Disease due to Meckel's diverticulum commonly rises during childhood and is rarely seen in adult life. Fistula formation between the urinary bladder is common with colonic diverticular disease but this complication from an inflamed Meckel's diverticulum is recorded in only six cases in the English literature. This complication of Meckel's diverticulum has often presented a challenge in both diagnosis and treatment.

## Patient and observation

**Figure 1:**
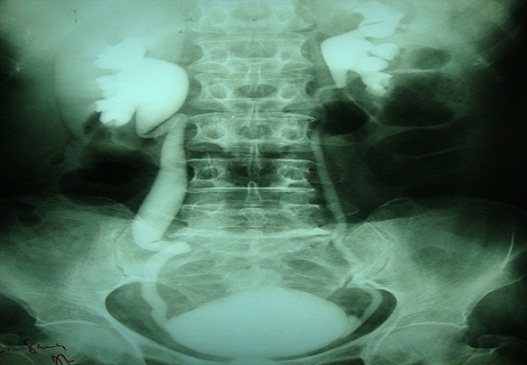
Excretory urogram control film: homogenous dilatation of the urinary right upper tract

**Figure 2:**
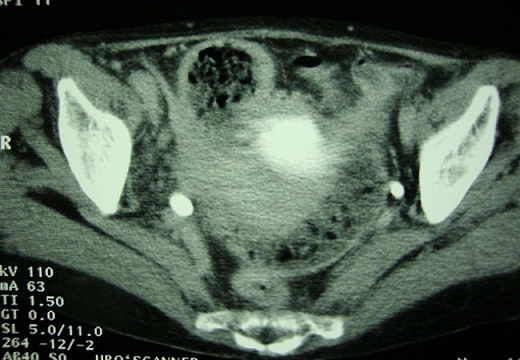
CT scan: small bowel segment adherent to the bladder with feces sign

A 66-year-old woman was admitted for fever (38° C) and dysuria of insidious onset, with no pneumaturia, she had episodes of abdominal distension, nausea and vomiting. Abdominal examination revealed mild suprapubic tenderness only. Vaginal examination was normal. The rest of the examination was normal. Laboratory investigations including urine microscopy, culture and sensitivity were normal. Colonoscopy and multiple biopsies were normal. Barium studies showed a thickened stenotic terminal ileum, a normal cecum and colon, and no evidence of perforation, external filling defects, or fistulisation. Excretory urogram control film demonstrated an homogenous dilatation of the urinary right upper tract (Figure 1), CT scan showed a small bowel segment adherent to the bladder with feces sign (Figure 2). Cystoscopy demonstrated a 3 cm. mass protruding from the upper posterior wall of the bladder and marked oedema of the overlying mucosa.

Laparotomy was done. Two feet from the ileocecal junction on the antimesenteric border of the ileum was a 3 cm long x 1 cm wide Meckel diverticulum adherent to the dome of the bladder. The diverticulum felt normal (no clinical evidence of ectopic mucosa) and a terminal ileectomy, a diverticulectomy were performed including a cuff of bladder encompassing the vesical end of the fistula, and an end-to-end ileal anastomosis was performed. An uneventful postoperative course followed and the patient was discharged asymptomatic. Histology revealed no evidence of gastric or pancreatic tissue in the diverticulum. There was evidence of mild Meckel diverticulitis and a sharp junction existed between the ileal and vesical mucosa.

## Discussion

Meckel's diverticulum is the most common congenital malformation of the gastrointestinal tract, it occurs in 0.8-4% of the population [1]. It can cause complications as ulceration, haemorrhage, intussusception, intestinal obstruction, perforation, these complications are estimated to occur in 4.2% or less of the cases [2]. Enterovesical fistulas usually result from diverticulitis, Crohn′s disease, or colorectal cancer [3, 4]. A perforated, or an inflamed Meckel′s diverticulum can also result in a vesico-diverticulum fistula, as noted in six previously reported cases [5–10]. The fistula is initiated by ulceration and inflammation of the bowel wall with subsequent perforation and extramural suppuration from the diverticulum. In our case, there was no coexisting bowel or bladder disease occurring with this vesico-diverticular fistula, unlike two reported cases with Crohn's ileitis [5, 8], a case where the vesicoenteric fistula was created by an ingested foreign body in Meckels diverticulum [10], and a case where the perforation was secondary to an enterolith [9]. Urinary symptoms predominate in the clinical picture of vesicoenteric fistula, then we confined chronic urinary tract infection, terminal pneumaturia, and fecaluria as the most common symptoms. Imaging techniques used for diagnosis include MRI (or even computerized tomography), which is highly effective in outlining the presence of gas in the bladder and thickening of the bladder wall in continuity with bowel segments. Cystoscopy also has a key role although visualization of the fistula is reported to vary from 6.7% to 67% [3]. This wide variation is probably due to the presence of significant submucosal erythema, associated with marked mucosal oedema and hyperplasia, which may hide the orifice of small fistulas. Nevertheless, the diagnosis was only made during the laparotomy, in all cases reported, with complete cure effected by excision of the diverticulum and closure of the bladder defect.

## Conclusion

Formation of a fistula from a Meckel's diverticulum to the bladder is extremely rare and may not be recognized. Only six cases are reported in the English literature, the diagnosis was always made during the laparotomy.
